# Comparison of vonoprazan and proton pump inhibitors for the treatment of gastric endoscopic submucosal dissection-induced ulcer: an updated systematic review and meta-analysis

**DOI:** 10.1186/s12876-024-03198-8

**Published:** 2024-03-15

**Authors:** Lizhen Chen, Dalei Jiang, Doudou Hu, Xianghua Cui

**Affiliations:** 1 Department of Infectious Disease, Qingdao Hospital, University of Health and Rehabilitation Sciences (Qingdao Municipal Hospital), Qingdao, Shandong China; 2 Department of Gastroenterology, Qingdao Hospital, University of Health and Rehabilitation Sciences (Qingdao Municipal Hospital), Qingdao, Shandong China

**Keywords:** Vonoprazan, Proton pump inhibitors, Endoscopic submucosal dissection, Ulcer, Meta-analysis

## Abstract

**Background:**

Both vonoprazan and proton pump inhibitors (PPIs) are currently used to treat artificial ulcers after gastric endoscopic submucosal dissection. However, evidence-based medicine proving the efficacy of vonoprazan is still lacking. Therefore, this meta-analysis aimed to compare the efficacy of vonoprazan and PPIs for the treatment of artificial ulcers after gastric endoscopic submucosal dissection.

**Methods:**

The PubMed, EMBASE and Cochrane Library databases were searched up to September 2023 for related randomized controlled trials (RCTs). RCTs that compared the efficacy of vonoprazan and PPIs in treating artificial gastric ulcers after gastric endoscopic submucosal dissection were included. Two independent reviewers screened the included studies, extracted the data and assessed the risk of bias. The following outcomes were extracted for comparison: ulcer healing rate, ulcer shrinkage rate, delayed postoperative bleeding rate, and ulcer perforation rate.

**Results:**

Nine randomized controlled trials involving 926 patients were included. The pooled results showed that vonoprazan had a significantly lower rate of delayed postoperative bleeding than did PPIs (RR = 0.46; 95% CI = 0.23–0.91; *P* = 0.03). No significant differences were found in terms of ulcer healing, shrinkage rates, or ulcer perforation rates between vonoprazan and PPIs.

**Conclusions:**

Compared with PPIs, vonoprazan is superior at reducing delayed postoperative bleeding after endoscopic submucosal dissection. However, further studies are needed to prove the efficacy of vonoprazan.

**Systematic Review Registration:**

Identifier CRD42024509227.

**Supplementary Information:**

The online version contains supplementary material available at 10.1186/s12876-024-03198-8.

## Background

Endoscopic submucosal dissection (ESD) has become the established treatment for early gastric cancer [1–3]. Unfortunately, ESD can cause artificial ulceration, which is occasionally linked to delayed postoperative bleeding and even perforation [4, 5]. Proton pump inhibitors (PPIs) are generally prescribed after ESD to inhibit the secretion of gastric acid and to promote the healing of iatrogenic ulcers [6–8].

Recently, vonoprazan, a novel acid inhibitor, has been used after ESD. As an active potassium-competitive acid blocker (P-CAB), vonoprazan inhibits gastric acid secretion in a K+-competitive and reversible manner [9–11] and reportedly has a more rapid, stronger and longer-lasting acid inhibitory effect than PPIs [11, 12]. Furthermore, vonoprazan is not affected by mealtimes or by CYP2C19 polymorphism [13, 14]. These findings indicate that vonoprazan may have a similar or better effect than PPIs have on the healing of ESD-induced ulcers.

Several studies [15–17] and meta-analyses [18, 19] have been performed to compare the efficacy of vonoprazan and PPIs for treating post-ESD artificial ulcers. However, evidence-based medicine proving the efficacy of vonoprazan and PPIs is still lacking. Whether vonoprazan is superior to PPIs remains controversial. For example, the meta-analysis by Kang et al. [18] showed no substantial difference in ulcer healing between vonoprazan and PPIs, while the meta-analysis by Liu et al. [19] noted that vonoprazan had better efficacy in ulcer healing than did PPIs. Therefore, we conducted an updated systematic review and meta-analysis to compare the efficacy of vonoprazan and PPIs in treating ESD-induced artificial ulcers.

## Methods

This meta-analysis was performed in accordance with a registered protocol (CRD42024509227).

### Inclusion criteria

Studies were included if they met the following inclusion criteria: (1) target population: patients who underwent ESD, (2) intervention: vonoprazan versus PPIs, and (3) methodological criteria: randomized controlled trials (RCTs). Case reports, case series, and review articles were excluded.

### Search strategy

Two authors (C.L. and J.D.) independently screened databases, including MEDLINE, EMBASE, and the Cochrane Collaboration Library, up to September 2023 for relevant studies. We used the search terms “vonoprazan”, “P-CAB”, “TAK-438”, “potassium-competitive”, “proton pump inhibitor”, “PPIs”, “endoscopic submucosal dissection” and “ESD”, with combinations of the operators “OR”, “AND” and “NOT”.

### Quality assessment

The quality of the included RCTs was independently assessed by two authors (J, D. and H. D.). Disagreements were resolved after discussion with another author (C. X.). For the included RCTs, we used the 12 criteria and instructions recommended by the Cochrane Back Review Group [18] for quality assessment.

### Data extraction

Two authors (C.L. and J.D.) extracted the data from the included studies independently. The general characteristics of each study were collected, namely, year of publication, author, study design, sample size, duration of follow-up, and patient characteristics. The following outcomes were extracted for comparison: ulcer healing post-ESD at 4 and 8 weeks, shrinkage rate at 4 and 8 weeks post-ESD, delayed postoperative bleeding, ulcer perforation, and adverse events (AEs). The follow-up time for AEs was 4 or 8 weeks post-ESD.

### Data analysis

Data analysis and synthesis were performed using Review Manager version 5.3 (Cochrane Collaboration). Continuous outcomes are expressed herein as the mean difference (MD) and 95% confidence interval (CI), and dichotomous outcomes are expressed as the risk ratio (RR) and 95% CI. The statistical heterogeneity among the included studies was evaluated using the χ2 test. *P* < 0.10 or I^2^ > 50% indicated substantial heterogeneity. Heterogeneous data were evaluated by a random-effects model [20]; otherwise, a fixed-effects model was used [21]. *P* < 0.05 was considered to indicate a statistically significant difference.

## Results

### Literature search

**Fig. 1:**
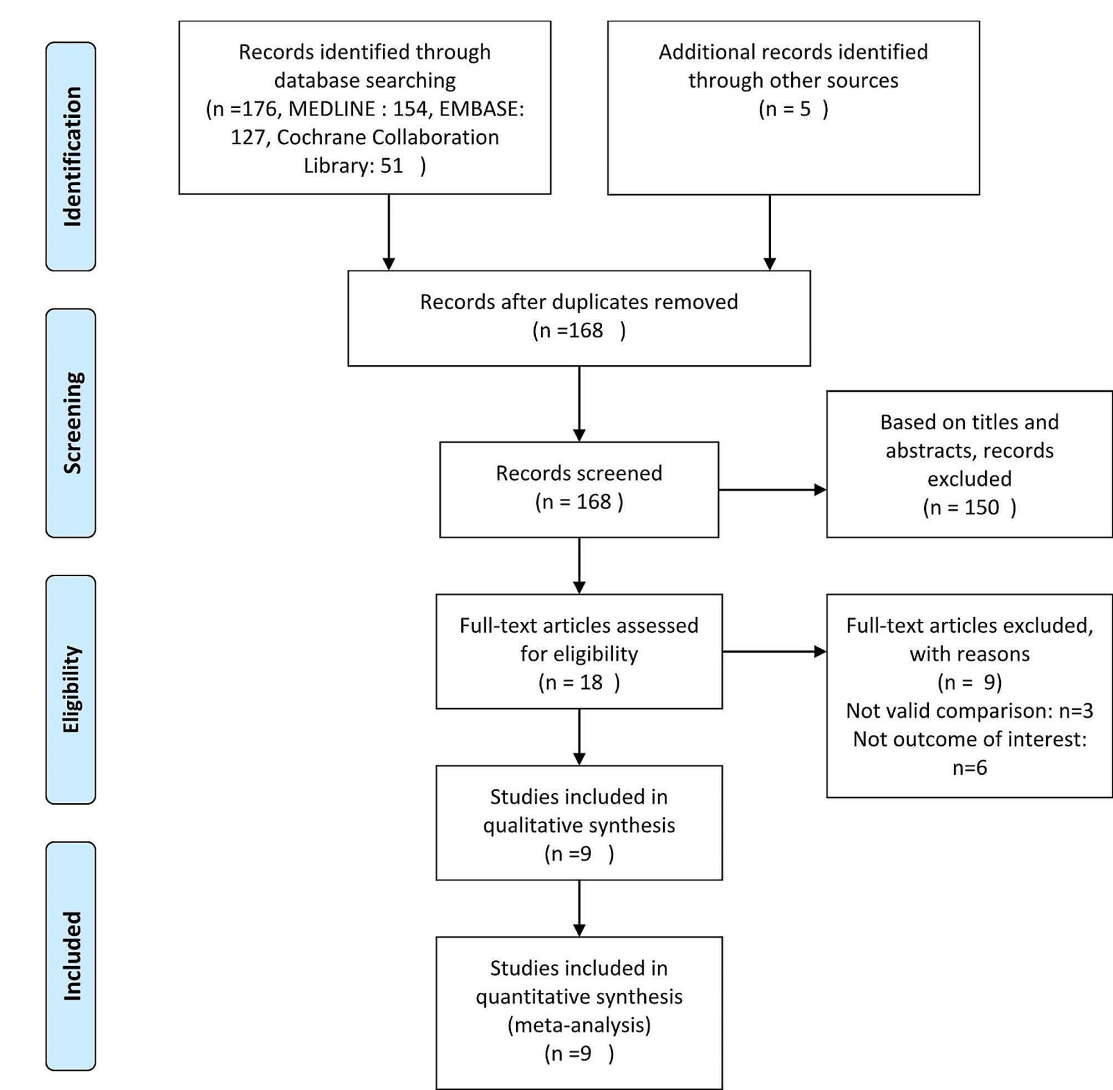
Flow of studies through review. *From:* Moher D, Liberati A, Tetzlaff J, Altman DG, The PRISMA Group (2009). *Preferred Reporting Items* for Systematic Reviews and Meta-Analyses: The PRISMA Statement. PLoS Med 6(7): e1000097. 10.1371/journal.pmed1000097. For more information, visit https://www.prisma-statement.org

A total of 176 articles that could potentially be included in this meta-analysis were identified. Of these articles, 150 were excluded after briefly screening the title, abstract, or full text. Ultimately, nine RCTs [15–17, 22–27] were included for analysis in this study. The retrieval flow diagram is displayed in Fig. 1.

### Study characteristics

Nine RCTs comparing the efficacy of vonoprazan and PPIs for managing ulcers post-ESD were included in the meta-analysis. The sample sizes of the nine RCTs ranged from 26 to 196. Overall, 470 patients in the vonoprazan group and 456 in the PPI group were included in this meta-analysis. Patients in the vonoprazan group received 20 mg vonoprazan or 20 mg vonoprazan plus 300 mg rebamipide daily. Patients in PPIs group were given 30 mg lansoprazole, 20 mg esomeprazole, 10 mg rabeprazole, or 20 mg esomeprazole plus 300 mg rebamipide daily. In the perioperative period, all patients received intravenous PPIs for one [27] or two [15–17, 23] days in five studies [15–17, 23, 27]. Then oral vonoprazan or oral PPIs were taken for 4 or 8 weeks. In the other four studies [22, 24–26], only oral vonoprazan or oral PPIs were taken by patients in the perioperative and follow-up periods. The main characteristics of the included studies are summarized in Table 1.

**Table 1:** Baseline characteristics of the studies included

Study	Follow-up	Country	VPZ group	PPI group	Location of tumor (U/M/L)	Endoscopic knives
Takahashi K 2016(15)	4w	Japan	VPZ (20 mg/d) 4w (14patients, age(yr) 71.9 ± 7.9, M/F 12/2)	Lansoprazole(30 mg/d) 4w (12patients, age(yr) 74.8 ± 8.3, M/F 10/2)	VPZ: 1/5/8 PPI: 0/4/8	Hook Knife (Olympus); Dual Knife (Olympus)
Tsuchiya I 2017(16)	8w	Japan	VPZ (20 mg/d) 8w (39patients, age(yr) 73, M/F 27/12)	Esomeprazole(20 mg/d) 8w (41patients, age(yr) 74, M/F 30/11)	VPZ: 9/13/19 PPI: 5/15/19	Triangle Tip Knife (KD-640 L; Olympus)
Hirai A 2018(17)	8w	Japan	VPZ (20 mg/d) 8w (74patients, age(yr) 73.16 ± 7.48, M/F 62/12)	Lansoprazole(30 mg/d) 8w (75patients, age(yr) 69.93 ± 11.0, M/F 55/20)	VPZ: 9/27/41 PPI: 4/29/42	Needle knife (KD-1 L; Olympus); IT Knife2 electrosurgical knife (KD-611 L; Olympus)
Ichida T 2019(22)	8w	Japan	VPZ (20 mg/d) + Rebamipide(300 mg/d) 8w (43patients, age(yr) 72.4, M/F 31/12)	Esomeprazole (20 mg/d) + Rebamipide(300 mg/d) 8w (39patients, age(yr) 73.9, M/F 34/5)	VPZ: 7/12/24 PPI: 4/18/17	Dual Knife (KD-650 L; Olympus)
Ishii Y 2018(23)	8w	Japan	VPZ (20 mg/d) + Rebamipide(300 mg/d) 8w (27patients, age(yr) 70, M/F 23/4)	Esomeprazole (20 mg/d) + Rebamipide(300 mg/d) 8w (26patients, age(yr) 70, M/F 22/4)	VPZ: 12/10/5 PPI: 14/10/2	IT knife2 (KD-611 L; Olympus); Dual Knife (KD-650U; Olympus)
Hamada K 2019(24)	8w	Japan	VPZ(20 mg/d) 8w (69patients, age(yr) 70.3 ± 6.8, M/F 51/18)	Lansoprazole(30 mg/d) 8w (70patients, age(yr) 70.1 ± 8.5, M/F 57/13)	body/antrum VPZ: 35/34; PPI: 34/36	Insulated-tipped knife-2 (Olympus); Flush Knife (Fuji Film Medical)
Komori H 2019(25)	4w	Japan	VPZ (20 mg/d) 4w (18patients, age(yr) 69 ± 9.3, M/F 13/5)	Rabeprazole(10 mg/d) 4w (15patients, age(yr) 70.9 ± 8.8, M/F 11/4)	VPZ: 1/4/13 PPI: 2/8/5	Dual knife (KD-650 L; Olympus); IT knife-2 (KD-611 L; Olympus)
Ban H 2021(26)	8w	Japan	VPZ (20 mg/d) 8w (101patients, age(yr) 71.5 ± 8.8, M/F 76/25)	Lansoprazole(30 mg/d) 8w (95patients, age(yr) 1.2 ± 8.6, M/F 69/26)	VPZ: 8/32/61 PPI: 10/39/46	Dual knife (KD-650; Olympus)
Kawai D 2021(27)	8w	Japan	VPZ (20 mg/d) 8w (85patients, age(yr) 73, M/F 63/22)	Lansoprazole (30 mg/d) 8w (83patients, age(yr) 73, M/F 58/25)	VPZ: 9/35/41 PPI: 11/42/30	Not applicable

Note: VPZ Vonoprazan, M/F Male/Female, U/M/L Upper/Middle/Lower

### Quality assessment

**Fig. 2:**
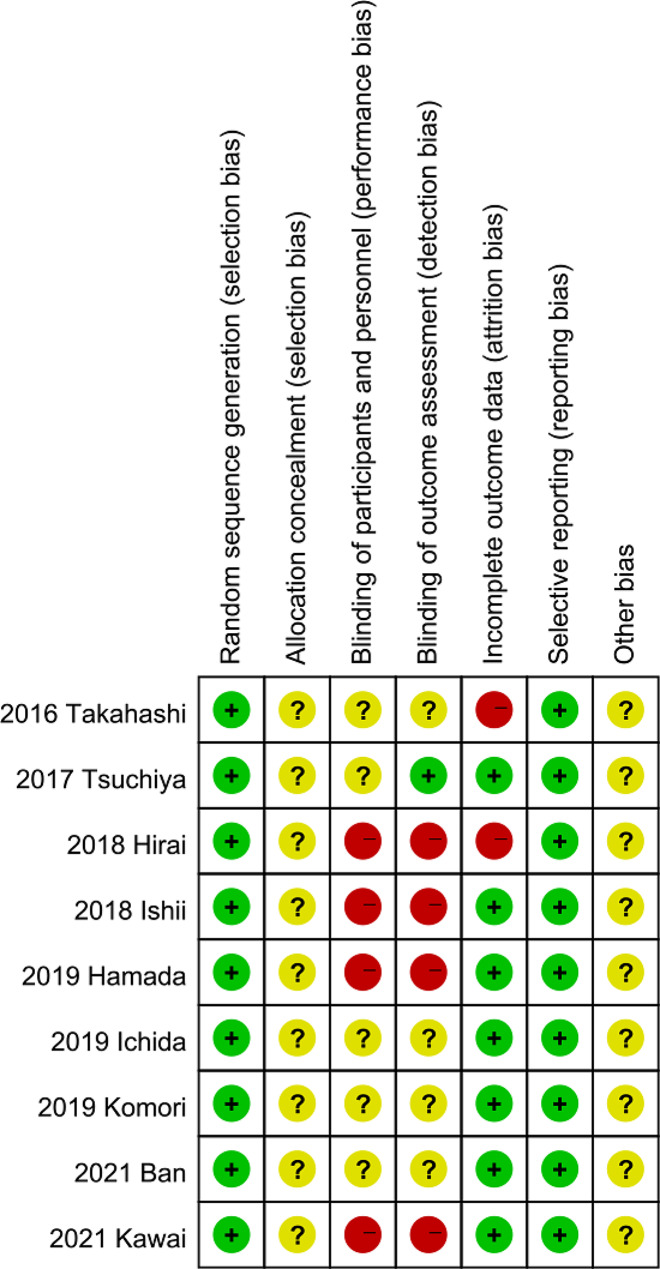
Quality assessment of the nine randomized controlled trials included

All the included nine studies had a randomized design. The quality of the included RCTs was assessed by the Cochrane assessment tool. The assessment of various items showed a medium risk of bias among the included studies (Fig. 2).

### Ulcer healing rate

#### Ulcer healing rate post-ESD at 4 weeks

**Fig. 3:**
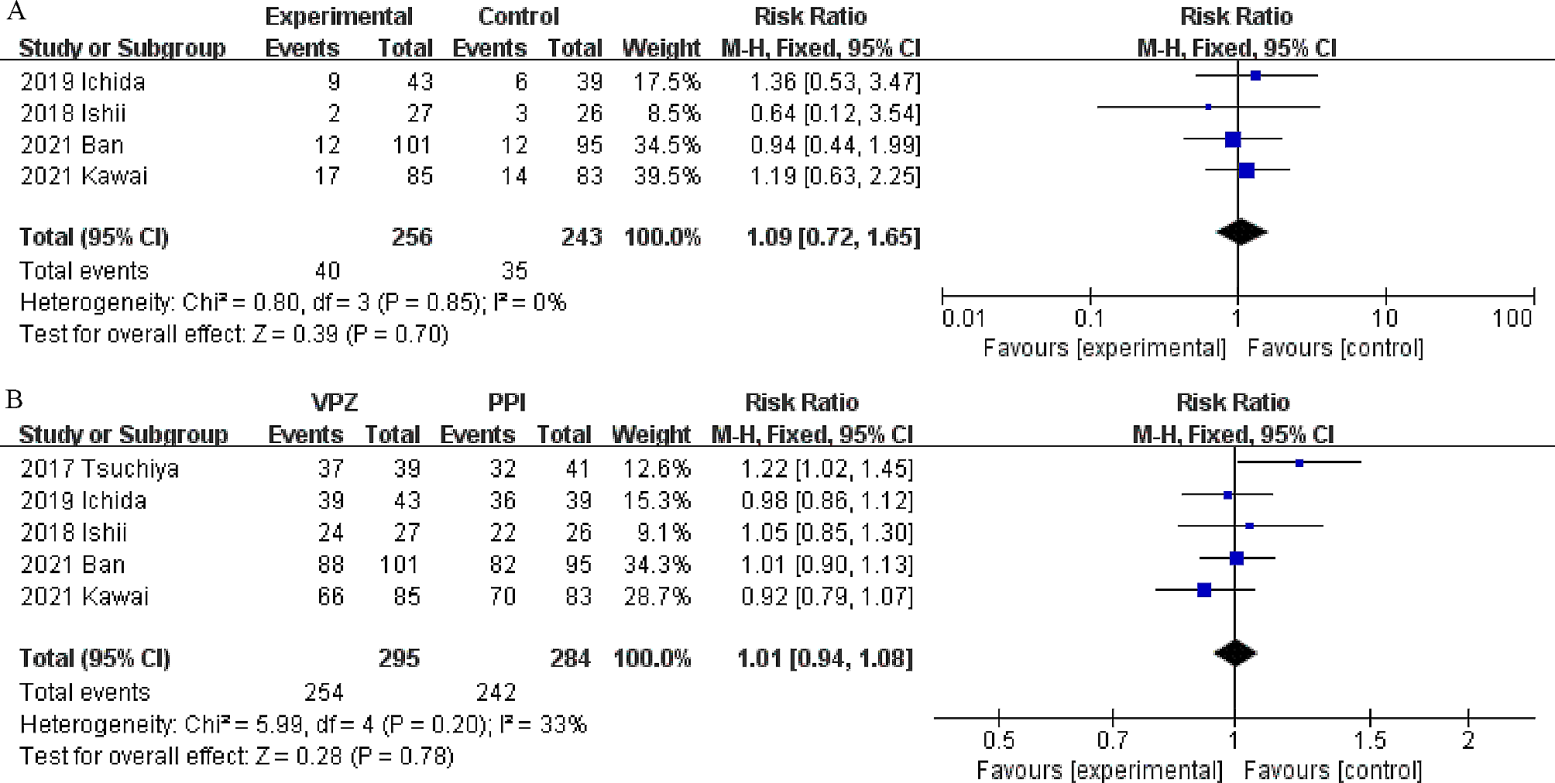
Forest plots of the ulcer healing rate at 4 weeks (**A**) and 8 weeks (**B**) in the vonoprazan and PPI groups

A total of four studies [22, 23, 26, 27] including 499 patients (256 patients in the vonoprazan group and 243 in the PPI group) reported ulcer healing post-ESD at 4 weeks. As depicted in Fig. 3A, there was no significant difference between the two groups in terms of the healing rate after ESD (RR 1.09, 95% CI 0.72–1.65, *P* = 0.70), and there was no significant heterogeneity (I^2^ = 0%) (Fig. 3A). Subgroup analysis revealed no significant difference between vonoprazan and lansoprazole (RR 1.07, 95% CI 0.66–1.74; *P* = 0.78) (Supplementary Fig. 1).

#### Ulcer healing post-ESD at 8 weeks

In all, 5 studies [16, 22, 23, 26, 27] including 579 patients (295 patients in the vonoprazan group and 284 in the PPI group) showed healing rate at 8 weeks post-ESD. No significant difference was found between the two groups (RR: 1.01, 95% CI: 0.94–1.08, *P* = 0.78; I^2^ = 33%) (Fig. 3B). Subgroup analysis revealed no significant difference between vonoprazan and lansoprazole (RR 0.97, 95% CI 0.89–1.06; *P* = 0.49) (Supplementary Fig. 2).

#### Delayed postoperative bleeding

**Fig. 4:**
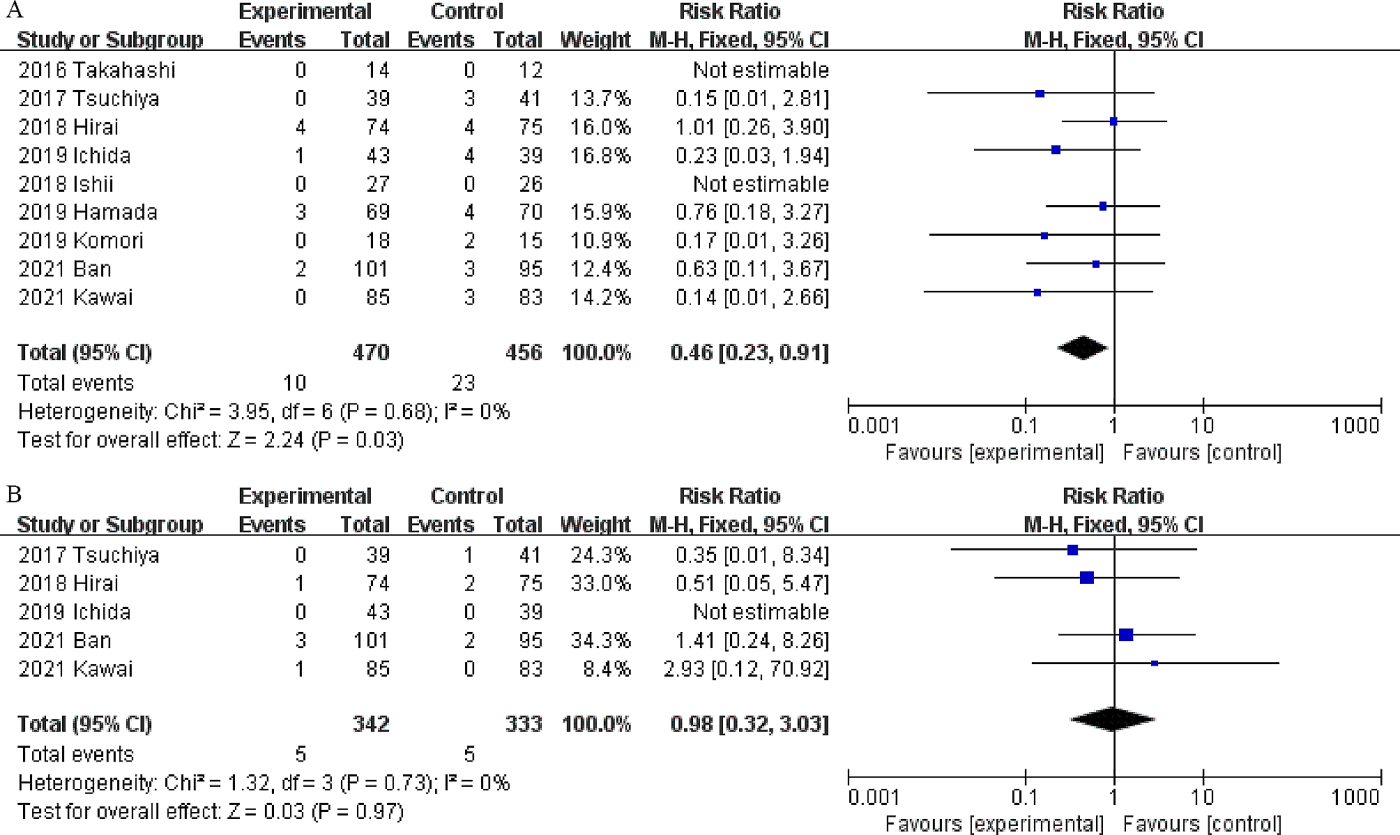
Forest plots of delayed postoperative bleeding (**A**) and ulcer perforation (**B**) rates in the vonoprazan and PPI groups

All nine studies, which included 926 patients (470 patients in the vonoprazan group and 456 in the PPI group), reported information about delayed postoperative bleeding complications. The incidence of delayed postoperative bleeding in the vonoprazan group was significantly lower than that in the PPI group (RR = 0.46, 95% CI = 0.23–0.91, *P* = 0.03; I^2^ = 0%) (Fig. 4A). Subgroup analysis revealed no significant difference between vonoprazan and lansoprazole (RR 0.65, 95% CI 0.29–1.45; *P* = 0.29) (Supplementary Fig. 3).

#### Ulcer perforation

**Fig. 5:**
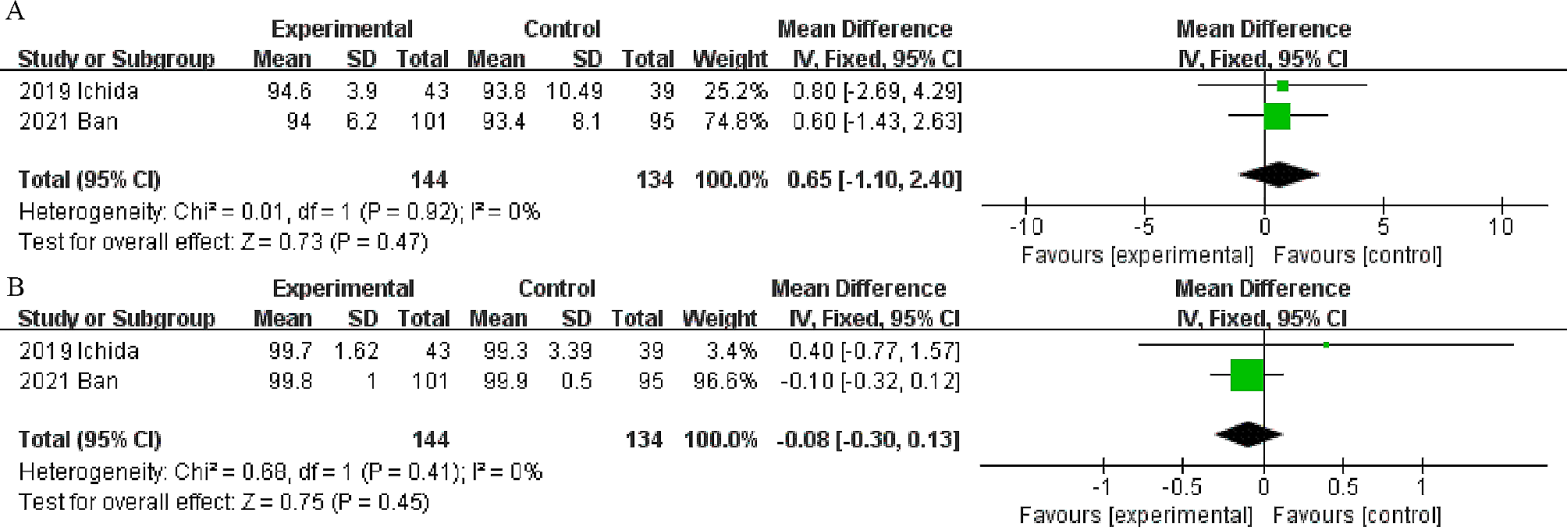
Forest plots of the ulcer shrinkage rate at 4 weeks (**A**) and 8 weeks (**B**) in the vonoprazan and PPI groups

A total of 5 studies [16, 17, 22, 26, 27] involving 675 patients reported ulcer perforation complications after ESD. As shown in Fig. 5, there was no significant difference in the ulcer perforation rate between the two groups according to the random effects model. (RR = 0.98, 95% CI = 0.32–3.03, *P* = 0.97; I^2^ = 0%) (Fig. 4B). Subgroup analysis revealed no significant difference between vonoprazan and lansoprazole (RR 1.19, 95% CI 0.34–4.09; *P* = 0.79) (Supplementary Fig. 4).

### Shrinkage rate

#### Shrinkage rate at 4 weeks post-ESD

Two studies [22, 26] including 278 patients reported differences in the shrinkage rate at 4 weeks post-ESD between the vonoprazan and PPI groups. The pooled results showed that there was no significant difference between the two groups in terms of the shrinkage rate at 4 weeks after ESD (MD 0.65, 95% CI -1.10-2.40; *P* = 0.47, I^2^ = 0%) (Fig. 5A).

#### Shrinkage rate at 8 weeks post-ESD

Two RCTs [22, 26] reported shrinkage rates at 8 weeks in the vonoprazan and PPI groups. No significant differences were found between the two groups (MD -0.08, 95% CI -0.3-0.13, *P* = 0.45; I^2^ = 0%) (Fig. 5B).

#### Adverse events

Seven studies [16, 17, 22, 24–27] with 847 patients (429 in the vonoprazan group and 418 in the PPI group) reported information about AEs. The pooled results showed that vonoprazan had a significantly lower rate of adverse events than did PPIs (RR = 0.54; 95% CI = 0.30–0.97; *P* = 0.04) (Supplementary Fig. 5).

## Discussion

Both vonoprazan and proton pump inhibitors (PPIs) are currently used to treat acid-related disorders, including artificial ulcers, after ESD [28–30]. Some studies have shown that vonoprazan is more effective than PPIs for healing artificial ulcers after ESD [31, 32]. On the other hand, some studies have shown that vonoprazan and PPIs are comparable in the treatment of ESD-induced ulcers [23, 26]. However, whether vonoprazan is superior to PPIs remains controversial. Therefore, a meta-analysis based on RCTs was conducted to clarify the effects of vonoprazan and PPIs on the healing of artificial ulcers.

According to our meta-analysis, there were no significant differences in the ulcer healing rate or shrinkage rate at 4 or 8 weeks between patients treated with vonoprazan or PPIs. Of the nine RCTs included in this meta-analysis, seven RCTs showed that vonoprazan was as efficacious as PPIs in the treatment of gastric ulcers after ESD. In contrast, the meta-analysis by Liu et al. [19] showed that vonoprazan had better efficacy in terms of ulcer shrinkage rates and healing. However, the strength of our meta-analysis is that all the included studies were RCTs, and two new well-designed studies [26, 27] were added. Our findings indicated that both vonoprazan and PPIs were adequate for the healing of artificial ulcers. Notably, artificial ulcers that occur after ESD develop in hypoacidic or normal environments and are relatively mild [33]. The gastric mucosal defense mechanisms are functioning, and inflammation is more localized [34]. Conversely, peptic ulcers occur in vulnerable locations with hyperacidity and extend deeper and laterally [33].

Delayed postoperative bleeding is the most common complication induced by ESD. Benites-Goñi et al. reported that delayed postoperative bleeding occurred in 5–7% of patients who underwent ESD [35]. Of the nine RCTs included in our meta-analysis, the rate of delayed postoperative bleeding in the vonoprazan group ranged from 0 to 5.4%. The included RCTs showed that vonoprazan had an equal or lower rate of post-ESD bleeding than did PPIs. Our meta-analysis showed that vonoprazan significantly reduced postoperative bleeding compared with PPIs. Similarly, Shiratori Y et al. [36] conducted a nationwide population-based study and reported that vonoprazan had a lower postoperative bleeding rate than did PPIs.

With respect to AEs, our meta-analysis showed that vonoprazan had a significantly lower rate of adverse events than did PPIs. In contrast, the meta-analysis conducted by Xu et al. [37] demonstrated that the incidence of adverse events was similar between vonoprazan and PPIs (*P* = 0.66). There are several differences between our meta-analysis and that of Xu et al. [37] Only RCTs were included in our meta-analysis, while RCTs and cohort and single-arm studies were included in the meta-analysis by Xu et al. [37] In addition, only patients with gastric ESD-induced ulcers were included in our meta-analysis, while patients with *H. pylori* infection, gastroesophageal reflux disease, peptic ulcer disease and ESD-induced ulcers were included in the meta-analysis by Xu et al. [37] Notably, only seven RCTs were included for the comparison of adverse events in our meta-analysis. Further study is needed to evaluate the safety of vonoprazan and PPIs.

There are several limitations in our study. First, the sample sizes of some of the trials included in this meta-analysis were small. Second, the patients included in this meta-analysis received different types and dosages of PPIs, which led to significant heterogeneity. The difference in the route of administration of PPIs might also lead to heterogeneity. As Ichida et al. [22] noted, their result of ulcer shrinkage rate was different to the study by Tsuchiya et al., which might be caused by the combination of oral and intravenous PPIs therapy in the latter study. On the other hand, A recent meta-analysis by Csiki et al. [38] showed that oral administration of PPIs was not inferior to the intravenous PPIs treatment in peptic ulcer bleeding after endoscopic management. So further studies are warranted. Third, all the trials included in this study were conducted in Japan, and thus, the results may not be applicable to other races.

## Conclusions

Based on this meta-analysis, vonoprazan is more effecative than PPIs are at reducing delayed postoperative bleeding from artificial ulcers after ESD, but there are no significant differences in ulcer healing, ulcer shrinkage rates, or ulcer perforation rates. Further analysis of additional trials is needed for the comparison of vonoprazan and PPIs in the treatment of artificial ulcers after ESD.

### Electronic supplementary material

Below is the link to the electronic supplementary material.

Supplementary Material 1

Supplementary Material 2

Supplementary Material 3

Supplementary Material 4

Supplementary Material 5

Supplementary Material 6

Supplementary Material 7

## Data Availability

All data relevant to the study are included in the article or uploaded as supplementary information.

## Abbreviations

ESD: Endoscopic submucosal dissection
PPIs: Proton pump inhibitors
P-CAB: Potassium-competitive acid blocker
RCTs: Randomized controlled trials
MD: Mean difference
CI: Confidence interval
RR: Risk ratio
AEs: Adverse events

## References

[1] Japanese Gastric Cancer Association (2021). Japanese gastric cancer treatment guidelines 2018 (5th edition). Gastric cancer: Official J Int Gastric Cancer Association Japanese Gastric Cancer Association.

[2] Katsuragi SY, Otsuki Y, Unno S, Kimata M, Yoshizawa Y, Tomatsu M, Shinmura K, Suzuki K, Sugimura H (2023). Evaluation of the widths of the mucosal strips in pathological examination of specimens of endoscopic submucosal dissection for early gastric cancer. Gastric cancer: Official J Int Gastric Cancer Association Japanese Gastric Cancer Association.

[3] Libânio D, Pimentel-Nunes P, Bastiaansen B, Bisschops R, Bourke MJ, Deprez PH, Esposito G, Lemmers A, Leclercq P, Maselli R (2023). Endoscopic submucosal dissection techniques and technology: European Society of Gastrointestinal Endoscopy (ESGE) Technical Review. Endoscopy.

[4] Gotoda T, Yamamoto H, Soetikno RM (2006). Endoscopic submucosal dissection of early gastric cancer. J Gastroenterol.

[5] Basford PJ, George R, Nixon E, Chaudhuri T, Mead R, Bhandari P (2014). Endoscopic resection of sporadic duodenal adenomas: comparison of endoscopic mucosal resection (EMR) with hybrid endoscopic submucosal dissection (ESD) techniques and the risks of late delayed bleeding. Surg Endosc.

[6] Yang Z, Wu Q, Liu Z, Wu K, Fan D (2011). Proton pump inhibitors versus histamine-2-receptor antagonists for the management of iatrogenic gastric ulcer after endoscopic mucosal resection or endoscopic submucosal dissection: a meta-analysis of randomized trials. Digestion.

[7] Ono H, Yao K, Fujishiro M, Oda I, Nimura S, Yahagi N, Iishi H, Oka M, Ajioka Y, Ichinose M (2016). Guidelines for endoscopic submucosal dissection and endoscopic mucosal resection for early gastric cancer. Dig Endoscopy: Official J Japan Gastroenterological Endoscopy Soc.

[8] Pimentel-Nunes P, Dinis-Ribeiro M, Ponchon T, Repici A, Vieth M, De Ceglie A, Amato A, Berr F, Bhandari P, Bialek A (2015). Endoscopic submucosal dissection: European Society of Gastrointestinal Endoscopy (ESGE) Guideline. Endoscopy.

[9] Andersson K, Carlsson E (2005). Potassium-competitive acid blockade: a new therapeutic strategy in acid-related diseases. Pharmacol Ther.

[10] Arikawa Y, Nishida H, Kurasawa O, Hasuoka A, Hirase K, Inatomi N, Hori Y, Matsukawa J, Imanishi A, Kondo M (2012). Discovery of a novel pyrrole derivative 1-[5-(2-fluorophenyl)-1-(pyridin-3-ylsulfonyl)-1H-pyrrol-3-yl]-N-methylmethanamine fumarate (TAK-438) as a potassium-competitive acid blocker (P-CAB). J Med Chem.

[11] Sakurai Y, Mori Y, Okamoto H, Nishimura A, Komura E, Araki T, Shiramoto M (2015). Acid-inhibitory effects of vonoprazan 20 mg compared with esomeprazole 20 mg or rabeprazole 10 mg in healthy adult male subjects–a randomised open-label cross-over study. Aliment Pharmacol Ther.

[12] Ashida K, Sakurai Y, Hori T, Kudou K, Nishimura A, Hiramatsu N, Umegaki E, Iwakiri K (2016). Randomised clinical trial: vonoprazan, a novel potassium-competitive acid blocker, vs. lansoprazole for the healing of erosive oesophagitis. Aliment Pharmacol Ther.

[13] Kagami T, Sahara S, Ichikawa H, Uotani T, Yamade M, Sugimoto M, Hamaya Y, Iwaizumi M, Osawa S, Sugimoto K (2016). Potent acid inhibition by vonoprazan in comparison with esomeprazole, with reference to CYP2C19 genotype. Aliment Pharmacol Ther.

[14] Hori Y, Matsukawa J, Takeuchi T, Nishida H, Kajino M, Inatomi N (2011). A study comparing the antisecretory effect of TAK-438, a novel potassium-competitive acid blocker, with lansoprazole in animals. J Pharmacol Exp Ther.

[15] Takahashi K, Sato Y, Kohisa J, Watanabe J, Sato H, Mizuno K, Hashimoto S, Terai S (2016). Vonoprazan 20 mg vs lansoprazole 30 mg for endoscopic submucosal dissection-induced gastric ulcers. World J Gastrointest Endoscopy.

[16] Tsuchiya I, Kato Y, Tanida E, Masui Y, Kato S, Nakajima A, Izumi M (2017). Effect of vonoprazan on the treatment of artificial gastric ulcers after endoscopic submucosal dissection: prospective randomized controlled trial. Dig Endoscopy: Official J Japan Gastroenterological Endoscopy Soc.

[17] Hirai A, Takeuchi T, Takahashi Y, Kawaguchi S, Ota K, Harada S, Kojima Y, Tominaga K, Tokioka S, Higuchi K (2018). Comparison of the effects of Vonoprazan and Lansoprazole for Treating Endoscopic Submucosal Dissection-Induced Artificial Ulcers. Dig Dis Sci.

[18] Kang H, Kim BJ, Choi G, Kim JG (2019). Vonoprazan versus proton pump inhibitors for the management of gastric endoscopic submucosal dissection-induced artificial ulcer: a systematic review with meta-analysis. Medicine.

[19] Liu C, Feng BC, Zhang Y, Li LX, Zuo XL (2019). The efficacy of vonoprazan for management of post-endoscopic submucosal dissection ulcers compared with proton pump inhibitors: a meta-analysis. J Dig Dis.

[20] DerSimonian R, Laird N (1986). Meta-analysis in clinical trials. Control Clin Trials.

[21] Mantel N, Haenszel W (1959). Statistical aspects of the analysis of data from retrospective studies of disease. J Natl Cancer Inst.

[22] Ichida T, Ueyama S, Eto T, Kusano F, Sakai Y (2019). Randomized Controlled Trial comparing the effects of Vonoprazan Plus Rebamipide and Esomeprazole Plus Rebamipide on gastric Ulcer Healing Induced by Endoscopic Submucosal Dissection. Intern Med (Tokyo Japan).

[23] Ishii Y, Yamada H, Sato T, Sue S, Kaneko H, Irie K, Sasaki T, Tamura T, Ikeda R, Fukuchi T et al. Effects of Vonoprazan Compared with Esomeprazole on the Healing of Artificial Postendoscopic Submucosal Dissection Ulcers: A Prospective, Multicenter, Two-Arm, Randomized Controlled Trial. Gastroenterology research and practice. 2018;2018:1615092. 10.1155/2018/1615092 PMC5835268 29670650

[24] Hamada K, Uedo N, Tonai Y, Arao M, Suzuki S, Iwatsubo T, Kato M, Shichijo S, Yamasaki Y, Matsuura N (2019). Efficacy of vonoprazan in prevention of bleeding from endoscopic submucosal dissection-induced gastric ulcers: a prospective randomized phase II study. J Gastroenterol.

[25] Komori H, Ueyama H, Nagahara A, Akazawa Y, Takeda T, Matsumoto K, Matsumoto K, Asaoka D, Hojo M, Yao T (2019). A prospective randomized trial of a potassium competitive acid blocker vs proton pump inhibitors on the effect of ulcer healing after endoscopic submucosal dissection of gastric neoplasia. J Int Med Res.

[26] Ban H, Inatomi O, Murata M, Otsuka T, Oi M, Matsumoto H, Bamba S, Andoh A (2021). Vonoprazan vs lansoprazole for the treatment of artificial gastric ulcer after endoscopic submucosal dissection: a prospective randomized comparative study. J Clin Biochem Nutr.

[27] Kawai D, Takenaka R, Ishiguro M, Okanoue S, Gotoda T, Kono Y, Takemoto K, Tsugeno H, Fujiki S (2021). Vonoprazan versus lansoprazole in the treatment of artificial gastric ulcers after endoscopic submucossal dissection: a randomized, open-label trial. BMC Gastroenterol.

[28] Ishida T, Dohi O, Yamada S, Yasuda T, Yamada N, Tomie A, Tsuji T, Horii Y, Majima A, Horie R (2021). Clinical outcomes of vonoprazan-treated patients after endoscopic submucosal dissection for gastric neoplasms: a prospective Multicenter Observation Study. Digestion.

[29] Yoshii S, Yamada T, Yamaguchi S, Hayashi Y, Nakahara M, Shibukawa N, Yamamoto M, Ishihara R, Kinoshita K, Egawa S (2020). Efficacy of vonoprazan for the prevention of bleeding after gastric endoscopic submucosal dissection with continuous use of antiplatelet agents. Endoscopy Int open.

[30] Yamamoto S, Takayama H, Shimodate Y, Takezawa R, Nishimura N, Doi A, Mouri H, Matsueda K, Mizuno M, Okada H (2020). Effect of Vonoprazan on delayed bleeding after endoscopic submucosal dissection for gastric neoplasia among antithrombotic drug users: a Single-Center, single-arm prospective observational Case Control Study. Acta Med Okayama.

[31] Maruoka D, Arai M, Kasamatsu S, Ishigami H, Taida T, Okimoto K, Saito K, Matsumura T, Nakagawa T, Katsuno T (2017). Vonoprazan is superior to Proton pump inhibitors in healing artificial ulcers of the stomach post-endoscopic submucosal dissection: a propensity score-matching analysis. Dig Endoscopy: Official J Japan Gastroenterological Endoscopy Soc.

[32] Yamasaki A, Yoshio T, Muramatsu Y, Horiuchi Y, Ishiyama A, Hirasawa T, Tsuchida T, Sasaki Y, Fujisaki J (2018). Vonoprazan is Superior to Rabeprazole for Healing Endoscopic Submucosal Dissection: Induced Ulcers. Digestion.

[33] Park HJ, Kim HS, Kim BR, Park SY, Hong JH, Jo KW, Kim JW (2013). Half-dose rabeprazole has an equal efficacy to standard-dose rabeprazole on endoscopic submucosal dissection-induced ulcer. Dig Dis Sci.

[34] Goto O, Fujishiro M, Kodashima S, Minatsuki C, Niimi K, Ono S, Yamamichi N, Koike K (2011). Short-term healing process of artificial ulcers after gastric endoscopic submucosal dissection. Gut Liver.

[35] Benites-Goñi H, Palacios-Salas F, Marín-Calderón L, Diaz-Arocutipa C, Piscoya A, Hernandez AV (2023). Short-term outcomes of endoscopic submucosal dissection for the treatment of superficial gastric neoplasms in non-asian countries: a systematic review and meta-analysis. Annals Gastroenterol.

[36] Shiratori Y, Niikura R, Ishii N, Ikeya T, Honda T, Hasatani K, Yoshida N, Nishida T, Sumiyoshi T, Kiyotoki S (2022). Vonoprazan versus proton pump inhibitors for postendoscopic submucosal dissection bleeding in the stomach: a multicenter population-based comparative study. Gastrointest Endosc.

[37] Xu W, Bai Z, Shang Y, Wang J, Wong Y, Qi X (2023). Incidence and type of adverse events in patients taking vonoprazan: a systematic review and meta-analysis. Therapeutic Adv Gastroenterol.

[38] Csiki E, Szabó H, Hanák L, Szakács Z, Kiss S, Vörhendi N, Pécsi D, Hegyi E, Hegyi P, Erőss B (2021). Oral Proton pump inhibitors may be as effective as intravenous in peptic ulcer bleeding: a systematic review and Meta-analysis. Clin Translational Gastroenterol.

