# Supplementary figures and images for: Comparison of vonoprazan and proton pump inhibitors for the treatment of gastric endoscopic submucosal dissection-induced ulcer: an updated systematic review and meta-analysis
Source: BMC Gastroenterol. 2024 Mar 15;24:110. doi: 10.1186/s12876-024-03198-8 (PMC10943859; doi:10.1186/s12876-024-03198-8)

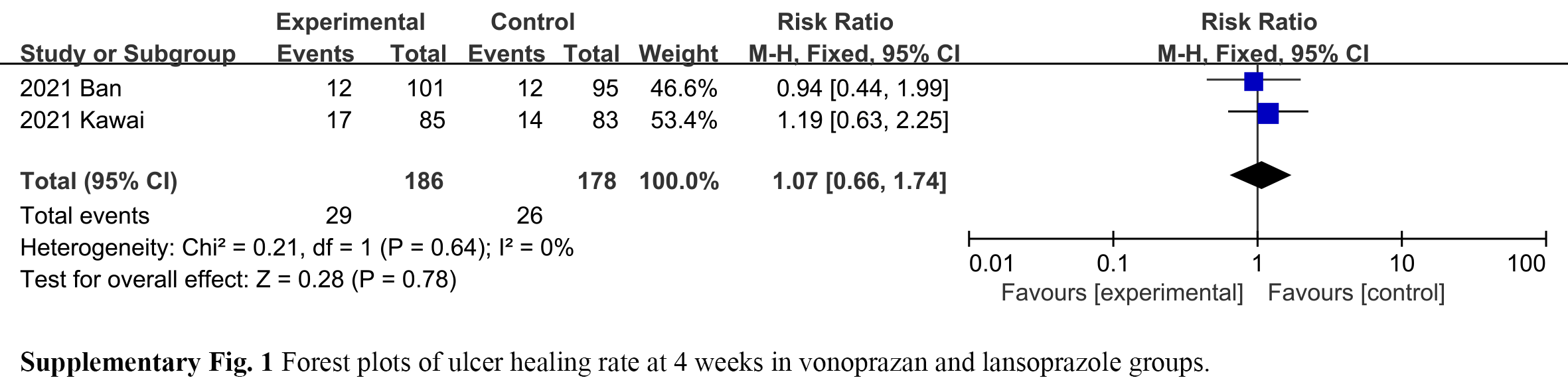

Supplement: Supplementary file 2 — Supplementary Material 2 [file 12876_2024_3198_MOESM2_ESM.tif]

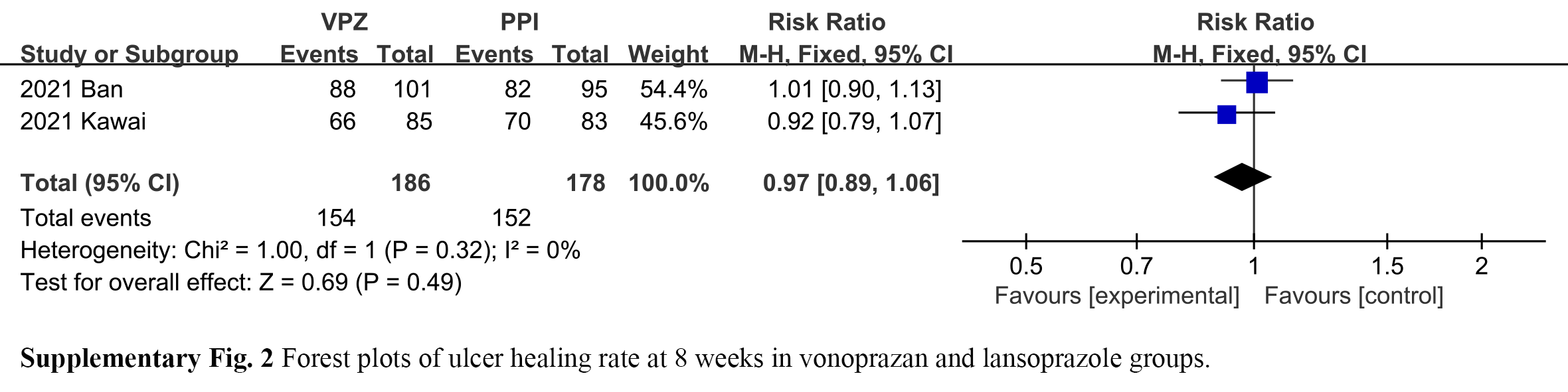

Supplement: Supplementary file 3 — Supplementary Material 3 [file 12876_2024_3198_MOESM3_ESM.tif]

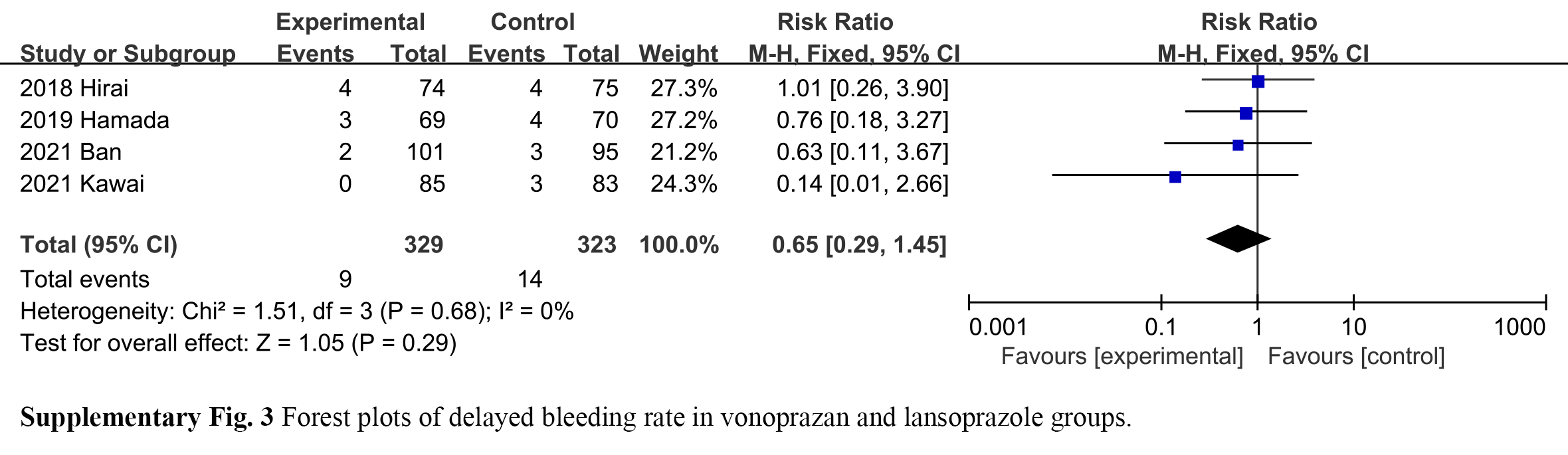

Supplement: Supplementary file 4 — Supplementary Material 4 [file 12876_2024_3198_MOESM4_ESM.tif]

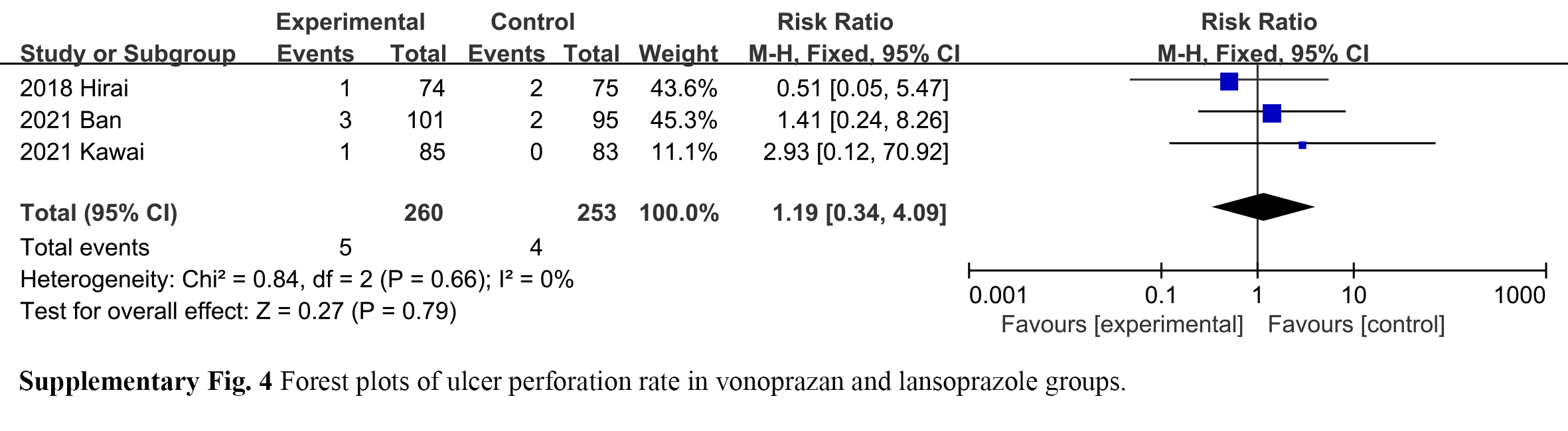

Supplement: Supplementary file 5 — Supplementary Material 5 [file 12876_2024_3198_MOESM5_ESM.tif]

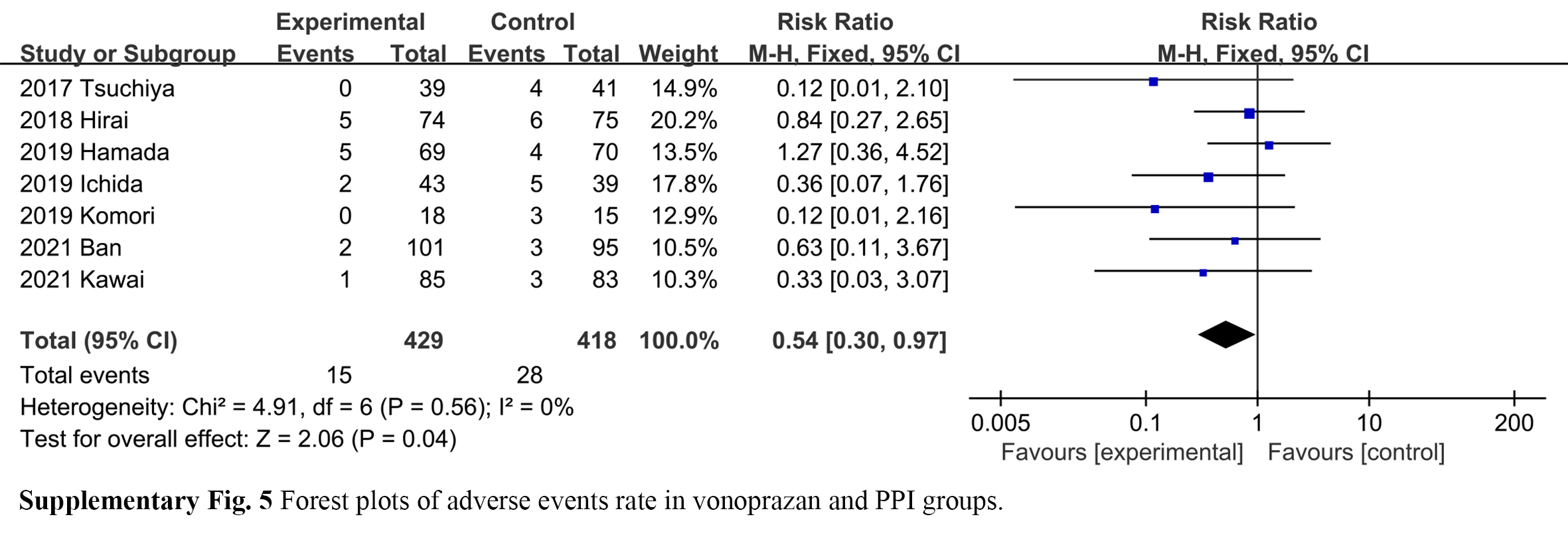

Supplement: Supplementary file 6 — Supplementary Material 6 [file 12876_2024_3198_MOESM6_ESM.tif]
