# Supplementary material for: Comparison of vonoprazan and proton pump inhibitors for the treatment of gastric endoscopic submucosal dissection-induced ulcer: an updated systematic review and meta-analysis
Source: BMC Gastroenterol. 2024 Mar 15;24:110. doi: 10.1186/s12876-024-03198-8 (PMC10943859; doi:10.1186/s12876-024-03198-8)
Supplement: Supplementary file 7 — Supplementary Material 7 [file 12876_2024_3198_MOESM7_ESM.docx]

**Supplementary Fig. 1** Forest plots of ulcer healing rate at 4 weeks in vonoprazan and lansoprazole groups.

**Supplementary Fig. 2** Forest plots of ulcer healing rate at 8 weeks in vonoprazan and lansoprazole groups.

**Supplementary Fig. 3** Forest plots of delayed bleeding rate in vonoprazan and lansoprazole groups.

**Supplementary Fig. 4** Forest plots of ulcer perforation rate in vonoprazan and lansoprazole groups.

**Supplementary Fig. 5** Forest plots of adverse events rate in vonoprazan and PPI groups.
